# Mitigating Job Burnout in Jordanian Public Healthcare: The Interplay between Ethical Leadership, Organizational Climate, and Role Overload

**DOI:** 10.3390/bs14060490

**Published:** 2024-06-11

**Authors:** Kayed Al’Ararah, Dilber Çağlar, Hasan Yousef Aljuhmani

**Affiliations:** 1 Business Management Department, Girne American University, North Cyprus Via Mersin 10, Kyrenia 99320, Turkey; 2 Faculty of Business and Economics, Girne American University, North Cyprus Via Mersin 10, Kyrenia 99320, Turkey; 3 Faculty of Business and Economics, Centre for Management Research, Girne American University, North Cyprus, Via Mersin 10, Kyrenia 99428, Turkey

**Keywords:** ethical leadership, organizational climate, role overload, employee well-being, job burnout, healthcare organizations

## Abstract

In today’s dynamic organizational landscape, characterized by rapid technological advancements and evolving workplace dynamics, understanding the factors influencing employee well-being is paramount. This study investigates the interplay between ethical leadership, organizational climate, role overload, and job burnout in public healthcare organizations across northern Jordan. By focusing on ethical leadership, organizational climate, and role overload as determinants of job burnout, this research provides insights into strategies for enhancing employee well-being. Drawing on ethical leadership theory, social exchange theory, and the job demands–resources model, this study employs PLS-SEM to analyze data collected from 260 employees working in Jordanian government hospitals. The findings reveal negative associations between ethical leadership and job burnout, highlighting the importance of ethical leadership behaviors in mitigating employee burnout. Additionally, a positive organizational climate is associated with lower levels of burnout, underscoring the impact of the broader organizational context on employee well-being. The study also explores the mediating role of organizational climate and the moderating effect of role overload in the relationship between ethical leadership and job burnout, providing insights into the complex dynamics at play in healthcare organizations. These findings enrich our understanding of the factors influencing employee well-being in healthcare contexts and underscore the importance of fostering ethical leadership and supportive organizational climates to mitigate job burnout.

## 1. Introduction

In contemporary organizational psychology, the role of ethical leadership in shaping employee well-being has become a subject of increasing interest and importance [1,2]. Brown and colleagues defined ethical leadership as “the demonstration of normatively appropriate conduct through personal actions and interpersonal relationships, and the promotion of such conduct to followers through two-way communication, reinforcement, and decision-making” ([3], p. 120). Leaders who prioritize ethical standards not only foster a positive organizational culture but also contribute to employee well-being by mitigating adverse psychological factors such as role overload, anxiety, and job exhaustion [4,5,6]. While the impact of ethical leadership on employee well-being has been acknowledged [7,8,9,10], understanding the underlying mechanisms remains a critical area of inquiry. Amidst these considerations, the phenomenon of job burnout has garnered significant attention, reflecting the deleterious consequences of prolonged stress and exhaustion within the healthcare workplace [11,12].

The scarcity of studies specifically examining the role of ethical leadership in promoting employee well-being within healthcare underscores the need for further investigation [13,14,15]. Promoting a favorable workplace climate and alleviating negative psychological outcomes, including job burnout, anxiety, and role overload, not only enhances organizational performance but also fosters creativity and innovation [16,17]. Recent research has highlighted the profound impact of workplace environment on employee well-being, particularly amidst challenges such as the COVID-19 pandemic [18,19]. Such findings underscore the importance of understanding the interplay between leadership, organizational context, and employee well-being in healthcare settings [20,21,22,23]. Ethical leadership, characterized by behaviors such as fairness, integrity, and concern for stakeholders, has emerged as a critical factor in shaping organizational culture and employee outcomes [3].

Ethical leadership has been shown to positively influence various workplace outcomes, including job satisfaction, organizational commitment, and performance [24,25]. However, the mechanisms through which ethical leadership affects employee well-being remain incompletely understood. One proposed pathway is through the promotion of positive workplace cultures that prioritize ethical behavior [13,26,27]. A culture that values ethics can create a supportive environment, reducing the occurrence of ethical conflicts and contributing to employee well-being [1]. Moreover, perceptions of ethical leadership may influence psychological outcomes such as perceived organizational support, further impacting employee well-being [22,28]. The organizational climate, representing the shared perceptions and attitudes of employees towards their work environment, serves as a contextual framework through which leadership behaviors manifest and influence individual experiences [29].

The theoretical foundation of this study is rooted in social exchange theory and the job demands–resources model. Social exchange theory proposes that interpersonal interactions in organizations involve the exchange of resources, where individuals respond to favorable treatment with increased commitment and performance [30]. Ethical leadership, characterized by transparency, trustworthiness, and moral integrity, promotes a positive exchange relationship between leaders and followers, thus enhancing employee engagement and well-being [3,9,25]. The job demands–resources model further explains how organizational factors, such as role overload, can affect the balance between job demands and resources, consequently influencing employee experiences of burnout [31,32,33,34]. By integrating these theoretical frameworks, this study aims to investigate how ethical leadership and organizational climate interact with role overload to alleviate or exacerbate job burnout among healthcare employees.

The motivation for this study arises from the urgent necessity to comprehend the intricate relationship between leadership, organizational context, and employee well-being in healthcare settings. As healthcare organizations endeavor to nurture ethical environments and boost employee engagement, a deeper understanding of the intricate dynamics of ethical leadership and its interaction with organizational climate and role demands is crucial for fostering robust and sustainable workplaces. By elucidating the moderating influence of role overload and the mediating effect of organizational climate through which ethical leadership influences job burnout among healthcare employees, this research aims to provide valuable insights for refining leadership approaches, shaping organizational policies, and developing intervention strategies geared toward alleviating job burnout and bolstering employee resilience amidst organizational pressures.

This study seeks to investigate the impact of ethical leadership on role overload and job burnout while exploring the mediating role of organizational climate. By examining these relationships in an Arabian context, specifically Jordan, this research aims to complement existing literature and verify results across diverse cultural settings [1]. The findings of this study hold implications for healthcare organizations aiming to enhance employee well-being, psychological outcomes, and ethical leadership practices in diverse cultural and organizational contexts. These findings have important implications for healthcare leaders and policymakers, emphasizing the importance of fostering ethical leadership practices and creating supportive organizational climates to promote employee well-being and mitigate job burnout.

## 2. Theoretical Framework and Hypotheses

### 2.1. Underpinning Theory

The underpinning theories guiding this study focus on social exchange theory (SET) and the job demands–resources (JD-R) model. Social exchange theory, rooted in sociology and psychology, provides a lens through which to understand interpersonal interactions within organizational contexts [30,35]. Central to social exchange theory is the notion that individuals engage in reciprocal exchanges of resources, such as trust, support, and recognition, within social relationships, including those with leaders and colleagues [36,37]. Within the workplace, ethical leadership behaviors, characterized by fairness, integrity, and concern for others, foster positive exchanges between leaders and followers, thereby cultivating a climate of trust and cooperation [3,9,25]. Employees reciprocate ethical leadership with increased commitment, engagement, and performance, contributing to a supportive organizational climate [17].

The JD-R model supplements SET by addressing the broader organizational context in which employee well-being and burnout are situated [38]. This model suggests that employees encounter various job demands, including workload, time pressure, and role ambiguity, necessitating physical, cognitive, and emotional resources for effective management [33,34]. Additionally, employees may benefit from job resources such as autonomy, social support, and opportunities for growth, which serve as buffers against the adverse effects of job demands and promote well-being [31,32,39]. Role overload, a prevalent stressor in contemporary work environments characterized by excessive task demands and limited resources, represents a crucial component of job demands that can contribute to employee burnout [40,41].

By integrating insights from SET and the JD-R model, this study endeavors to shed light on the complex interconnections among ethical leadership, organizational climate, role overload, and job burnout. Specifically, it seeks to clarify how ethical leadership behaviors influence the quality of social exchanges and organizational climates, thereby shaping employees’ perceptions of job demands and resources. Furthermore, the research investigates the moderating influence of role overload on the relationship between ethical leadership, organizational climate, and job burnout. Through the exploration of these relationships using theoretical frameworks rooted in social science, the study aims to provide a nuanced comprehension of the mechanisms underlying employee well-being and to offer practical implications for organizational leaders and practitioners striving to cultivate healthier work environments.

### 2.2. Ethical Leadership

Ethical leadership has emerged as a pivotal construct in contemporary organizational research, emphasizing the significance of ethics and values in guiding leadership behaviors [13,21]. Drawing on social learning theory, Brown and colleagues conceptualize ethical leadership as “the demonstration of normatively appropriate conduct through personal actions and interpersonal relationships” and embodies principles such as integrity, fair treatment, and ethical standards ([3], p. 120). This form of leadership is distinct from other leadership styles, such as transformational leadership, which focuses on inspiring and motivating followers to achieve extraordinary outcomes through vision, communication, and change [42]. While transformational leadership can encompass ethical behaviors, it is not synonymous with ethical leadership [43,44].

Ethical leaders integrate moral principles into their beliefs, values, and behaviors, promoting a culture of integrity and accountability within organizations [25,45]. Brown et al. [3] established a comprehensive ethical leadership scale that highlights the role of leader behavior in organizational outcomes. According to Kanungo [46], ethical leaders prioritize the well-being of others and abstain from behaviors that could cause harm. They are perceived as trustworthy and engage employees by considering their participation in decision-making processes [47].

Social learning theory posits that employees observe and emulate the ethical behaviors demonstrated by their leaders [3]. Furthermore, social exchange theory explains the relationship between ethical leaders and employee behaviors, suggesting that followers reciprocate the support and care received from their leaders with desirable work behaviors [30]. Ethical leaders focus on the long-term concerns of employees and treat them with respect, care, and dignity [48,49].

Empirical research has shown that ethical leadership significantly impacts employee well-being. Ethical leaders foster an environment where employees feel valued and supported, leading to improved job satisfaction and well-being [50,51]. They enhance job characteristics such as autonomy, role clarity, and job empowerment, which are positively related to employee well-being [40,52,53]. Acknowledgment and respect from ethical leaders also correlate positively with well-being [6,51,54,55,56].

Despite the abundance of research on the positive consequences of ethical leadership, there is a need to explore its role in enhancing both job burnout and broader psychological well-being [9,10,57], especially in the context of the healthcare [12,20,21,22,58]. This study aims to build on this foundation by examining the direct and indirect effects of ethical leadership on employee well-being within the context of public healthcare organizations.

By incorporating ethical leadership into organizational strategies, leaders can significantly influence employee well-being and mitigate the adverse effects of job burnout, thereby fostering a healthier, more productive workforce. This study aims to explore these dynamics within healthcare settings, providing insights into how ethical leadership can support employee well-being.

### 2.3. Job Burnout and Employee Well-Being in Healthcare

The dynamic nature of modern workplaces, especially in high-stress environments like healthcare, necessitates a comprehensive understanding of factors influencing employee well-being [22]. While previous studies have extensively explored job burnout, the broader concept of psychological well-being has often been overlooked, despite its critical importance in ensuring long-term employee health and productivity [59].

Psychological well-being encompasses more than the mere absence of burnout; it includes positive dimensions such as job satisfaction, emotional resilience, and overall mental health [60]. Job burnout research originated in the human services sector, driven by the need to understand the syndrome affecting overworked and emotionally exhausted workers [61]. Burnout is commonly conceptualized as a three-dimensional construct comprising emotional exhaustion, depersonalization, and reduced personal accomplishment [62]. Emotional exhaustion is the core dimension, characterized by the depletion of emotional resources, serves as a critical indicator of employee well-being [63]. As emotional exhaustion intensifies, it engenders negative work-related emotions and experiences, impeding work satisfaction and motivation, and compromising overall work performance [31,32,34,64]. Recognizing the centrality of employee well-being, organizations strive to foster positive work environments that mitigate emotional exhaustion and promote psychological health among employees. Therefore, it is crucial to integrate these broader aspects into our research model to provide a holistic view of employee well-being.

The coronavirus pandemic has significantly impacted individuals, organizations, and societies. Healthcare workers, in particular, have faced severe adverse effects on their subjective well-being due to the increased ethical challenges and stressors associated with managing the pandemic [22,65,66,67]. Subjective well-being, which relates to an individual’s personal experiences and perceptions, is critical in crisis situations like the COVID-19 pandemic, as it helps organizations maintain operations even during emergencies [68].

The COVID-19 pandemic has intensified these challenges, with healthcare workers facing unprecedented levels of stress and ethical dilemmas [69,70,71]. Healthcare organizations have increasingly focused on leadership processes that prioritize employees’ health and subjective well-being [22]. During the COVID-19 pandemic, organizational leaders have faced significant challenges in maintaining employees’ well-being [72]. Ethical leadership, defined as the extent to which a leader adheres to normatively appropriate behaviors [3], has been identified as a crucial supportive factor in mitigating the pandemic’s devastating impact [73].

Conversely, job burnout, a significant issue in the healthcare sector, results from chronic exposure to job stress and leads to emotional exhaustion, depersonalization, and reduced personal accomplishment [41,61]. Burnout negatively impacts workers’ psychological, physiological, and behavioral well-being, depleting personal resources and leading to fatigue, psychological erosion, and potentially harmful coping behaviors [62]. Addressing these challenges, especially in the context of the healthcare setting, underscores the importance of ethical leadership in mitigating burnout and enhancing overall well-being [20,28,58,74].

Moreover, the organizational climate plays a crucial role in shaping employees’ experiences and perceptions at work. A positive organizational climate, characterized by trust, openness, and support, can significantly enhance employees’ psychological well-being [75,76,77]. Conversely, role overload, which refers to excessive work demands placed on employees, is a known predictor of both burnout and reduced well-being [40,78,79]. By examining the moderating effects of role overload, this study seeks to provide a nuanced understanding of how work demands can impact the relationship between ethical leadership, organizational climate, and employee well-being.

**Figure 1:**
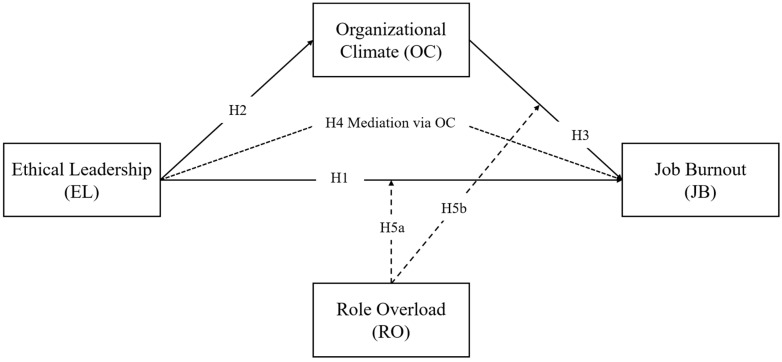
Research model.

In light of these considerations, this study aims to fill this gap by constructing a theoretical and empirical framework to elucidate the mediating effects associated with various outcomes of ethical leadership (see Figure 1). Through the integration of insights from organizational psychology literature and empirical evidence, this research endeavors to unravel the complexities of the relationship between ethical leadership and employee well-being, offering valuable insights for both organizational practice and theory development in healthcare settings.

### 2.4. Ethical Leadership and Job Burnout

Numerous studies in organizational psychology have underscored the negative ramifications of burnout, a phenomenon characterized by decreased job satisfaction, motivation, and performance stemming from prolonged occupational stress [11,34,80]. Burnout manifests as physical and mental exhaustion, accompanied by feelings of hopelessness and apathy [81]. Within the healthcare sector, particularly among nurses combating challenges such as the COVID-19 pandemic, burnout rates have surged, exacerbating emotional exhaustion and compromising overall well-being [18,19,82].

Ethical leadership, characterized by moral integrity and ethical decision-making, emerges as a potential buffer against the deleterious effects of burnout [40]. Ethical leaders guide their teams with transparency and fairness, fostering trust and respect within the organizational context [6,83]. They cultivate a positive work environment conducive to employee well-being, mitigating stressors and promoting psychological resilience [3,47,51,84]. Ethical leadership has been associated with reduced emotional exhaustion and depersonalization, components of burnout prevalent in healthcare settings [42,43,85].

In the healthcare sector, empirical evidence suggests that ethical leadership exerts a direct positive effect on employee well-being [20,22,86]. Employees under ethical leaders experience greater role clarity and reduced role overload, mitigating burnout risks and enhancing job satisfaction [40]. Ethical leadership fosters a sense of purpose and meaning at work, promoting trust and engagement among employees [83]. Conversely, unethical leadership practices engender cynicism and disengagement, exacerbating burnout and diminishing overall well-being [87].

Ethical leadership’s influence extends beyond the workplace, serving as a protective factor against burnout amidst challenging circumstances such as the COVID-19 pandemic [65]. Leaders who exhibit ethical behaviors create a supportive climate, enabling employees to cope effectively with stressors and maintain psychological well-being [28]. Taken together, ethical leadership emerges as a salient determinant of healthcare employees’ well-being, exerting a direct negative effect on job burnout [6,40,83]. By fostering a positive work environment, promoting trust, and cultivating meaningful relationships, ethical leaders mitigate burnout risks and enhance overall psychological health among employees [88]. As organizations navigate complex challenges and strive to promote employee well-being, ethical leadership practices offer a promising avenue for fostering resilience and enhancing organizational effectiveness. Thus, we expect that:

**H1.**  *Ethical leadership will be negatively related to employee job burnout.*

### 2.5. Ethical Leadership and Organizational Climate

Drawing from extensive literature and empirical evidence, the relationship between ethical leadership and organizational climate emerges as significant and multifaceted. Ethical leadership, characterized by traits such as moral integrity, honesty, and fairness, holds a pivotal role in shaping the overall atmosphere and dynamics within an organization [26,89]. Organizational climate, as defined by Schneider et al. [29], encompasses the collective perceptions of employees regarding the psychological, social, and physical characteristics of their work environment. It mirrors the prevailing norms, values, and expectations within the organization [16]. A positive organizational climate is distinguished by traits such as trust, open communication, collaboration, and mutual respect among employees, thereby fostering an environment conducive to productivity and job satisfaction [90,91,92].

Multiple studies have illuminated the positive correlation between ethical leadership and organizational climate [17,21]. Ethical leaders act as exemplars, demonstrating ethical principles and behaviors that establish the culture of the organization [93]. They emphasize transparency, fairness, and accountability in decision-making, fostering an environment where employees feel valued and respected [94]. Research in healthcare contexts has emphasized the advantageous impact of ethical leadership on organizational climate. Prior studies have underscored the significance of supportive and empowering leadership in cultivating a positive work environment and alleviating stress and burnout among healthcare professionals [75,95,96].

Furthermore, studies by Liu et al. [97] and Neilson and Munir [98] have demonstrated that ethical leadership enhances employee trust and cooperation, facilitating effective teamwork and communication within healthcare organizations. Ethical leaders create an environment where employees feel empowered to voice their opinions and concerns, fostering a culture of openness and inclusivity [93,99,100]. Moreover, ethical leadership has been associated with lower rates of workplace deviance and higher levels of job satisfaction among healthcare employees [26,53,76]. By promoting justice, transparency, and participatory decision-making, ethical leaders inspire commitment and engagement among healthcare employees, contributing to a positive organizational climate [101,102].

While the connection between ethical leadership and ethical climate has been thoroughly examined, there persists a necessity for additional research on the broader ramifications of ethical leadership on the overall organizational climate, particularly within healthcare settings [26,103]. This study aims to fill this void by examining the relationship between ethical leadership and organizational climate in a hospital setting, anticipating that ethical leadership exerts a positive influence on the organizational climate. By elucidating the mechanisms through which ethical leadership molds the workplace environment, organizations can gain a deeper understanding and capitalize on the influence of ethical leadership to nurture a culture characterized by integrity, collaboration, and excellence. Hence, we anticipate that:

**H2.**  *Ethical leadership will be positively related to organizational climate.*

### 2.6. The Mediating Role of Organizational Climate

The interplay between ethical leadership, organizational climate, and job burnout represents a complex dynamic within the workplace environment. Ethical leadership, distinguished by moral integrity, transparency, and fairness, has been demonstrated to exert a significant impact on employee well-being and organizational outcomes [26,89]. Conversely, organizational climate reflects the collective perceptions of employees concerning the psychological, social, and physical dimensions of their work environment [104]. It encompasses elements such as trust, communication, collaboration, and fairness, which collectively contribute to the overall atmosphere within the organization [16]. Job burnout, characterized by emotional exhaustion, depersonalization, and reduced personal accomplishment, is a prevalent issue across various workplace settings [34,80]. It is associated with adverse organizational outcomes, including diminished job satisfaction, decreased productivity, and heightened turnover rates [81].

In the healthcare setting, several studies have highlighted the relationship between ethical leadership and job burnout. Ethical leaders, by demonstrating integrity, promoting fairness, and fostering a supportive work environment, can mitigate the risk of burnout among employees [83,85]. Employees who perceive their leaders as ethical are more likely to experience greater job satisfaction, reduced stress levels, and lower rates of burnout [85,105]. Research has consistently demonstrated a negative correlation between ethical leadership and job burnout, with ethical leaders fostering a supportive work environment that mitigates the risk of burnout among healthcare employees [58,106,107,108].

Moreover, research has underscored the significance of organizational climate as a mediator in the connection between leadership behaviors and employee outcomes [17,92,109,110]. Organizational climate reflects the collective perceptions and experiences of employees concerning the work environment, influencing their attitudes, behaviors, and levels of job satisfaction [16,91]. A positive organizational climate, characterized by trust, open communication, and collaboration, can act as a protective factor against job burnout by cultivating a supportive and conducive work environment [26,77]. Consequently, it is hypothesized that organizational climate mediates the relationship between ethical leadership and job burnout. Ethical leaders shape the organizational climate by fostering trust, promoting fairness, and encouraging open communication, which subsequently contributes to reduced levels of job burnout among employees. Hence, we anticipate that:

**H3.**  *Positive organizational climate will be negatively related to employee job burnout.*

**H4.**  *Positive organizational climate will mediate the negative relationship between ethical leadership and employee job burnout.*

### 2.7. The Moderating Effect of Role Overload

Role overload has been defined as “situations in which employees feel that there are too many responsibilities or activities expected of them in light of the time available, their abilities, and other constraints” ([111], p. 741). It is a pervasive issue in contemporary work environments, driven by rapidly changing work dynamics and increasing job demands [112]. Role overload negatively impacts job performance and is associated with feelings of resource loss among employees [78,113].

The literature emphasizes the moderating role of role overload in the relationship between ethical leadership, organizational climate, and job burnout. Role overload acts as a situational constraint factor, impeding employees from leveraging their strengths for positive outcomes [114,115,116]. It is associated with adverse follower outcomes, such as decreased organizational commitment and heightened absenteeism [40,117]. Furthermore, role overload contributes to burnout, particularly in terms of emotional exhaustion [41]. However, the influence of ethical leadership on job burnout may vary depending on the extent of role overload experienced by employees [5,6,83,85]. In this regard, the presence of role overload can attenuate this relationship by amplifying stress and burden on employees [44,115,118,119,120]. Employees facing role overload may perceive ethical leadership behaviors as insufficient in alleviating job strain, thereby diminishing the positive impact of ethical leadership on well-being [1,13,40]. Consequently, ethical leaders must ensure manageable workloads to prevent role overload and promote employee well-being. Thus, we anticipate that:

**H5a.**  *Role overload will moderate the relationship between ethical leadership and job burnout.*

Organizational climate, characterized by trust, communication, and support, significantly influences employee experiences and outcomes [16,77,91,109]. A positive organizational climate fosters psychological safety and reduces the likelihood of job burnout among employees [121,122,123]. However, role overload moderates the relationship between organizational climate and job burnout, as employees facing high role overload may struggle to benefit fully from a positive climate [112,124]. Supportive workplace cultures, open communication, and clear expectations can help alleviate role overload and create a healthy work environment [77,102,125]. The relationship between workplace climate and job burnout is strongly influenced by role overload, with lower levels of role overload associated with supportive environments and clear expectations [126,127,128]. Therefore, the moderation impact of role overload underscores the importance of considering individual differences and contextual factors in understanding the relationship between organizational dynamics and employee well-being. Healthcare leaders should prioritize initiatives to improve workplace climate aspects to reduce job overload and boost employee satisfaction and well-being [12,40,129,130]. Encouraging teamwork, providing resources, and fostering open communication are essential steps toward mitigating role overload and promoting employee resilience in the face of job demands [6,116,131,132,133]. Thus, we expect that:

**H5b.**  *Role overload will moderate the relationship between organizational climate and job burnout.*

## 3. Research Design and Methodology

### 3.1. Sample and Procedure

The sampling procedure for this research involved targeting all employees within government hospitals situated in the northern region of Jordan, covering the governorates of Irbid, Jerash, Mafraq, and Ajloun. With a reported population of 4345 individuals based on the Ministry of Health in Jordan, comprising medical professionals and administrative staff, the study specifically focused on individuals working in public healthcare organizations (HCOs) in the northern or middle parts of Jordan. Following the methodologies of previous studies conducted by Azar et al. [134] and Hijazi [135] on Jordanian HCOs, a cross-sectional approach was adopted, with data collection occurring between July and September 2022. A non-probability convenience sampling method was utilized due to some HCOs not granting permission for survey distribution. Out of the 30 public hospitals identified across Jordan from the Ministry of Health’s website (https://www.moh.gov.jo), 13 public hospitals from 4 major cities in the northern region were chosen, of which 6 agreed to participate in the study. Participants were drawn from both medical and administrative staff. The inclusion criteria necessitated that participants hold at least a BA degree and work in public hospitals in the northern or middle parts of Jordan.

### 3.2. Data Collection

An online survey methodology was utilized, with the researcher obtaining approval from the public relations departments of identified organizations to facilitate the process. The survey link was disseminated to eligible personnel in establishments where approval was granted. To minimize respondent bias, a cover letter accompanied the survey, stressing the voluntary nature of participation, confidentiality, and the scientific purpose of the data collection [136]. The survey aimed to investigate various aspects related to employee well-being and organizational dynamics within the context of public healthcare organizations in the northern region of Jordan. Following the guidelines outlined by Sekaran and Bougie [137], 750 questionnaires were distributed to potential respondents to attain an appropriate sample size for the population under investigation. Among the collected responses, 263 were deemed valid, with 3 incomplete questionnaires excluded, resulting in a final sample size of 260 participants. Data analysis was conducted using Structural Equation Modeling (SEM), with the gathered data offering insights into the interrelationships among ethical leadership, organizational climate, role overload, and job burnout among employees in government hospitals in the northern region of Jordan.

### 3.3. Sample Profile

The respondent profile of the 260 final participants reflects a diverse demographic composition. As indicated in Table 1, the majority of respondents were male, comprising 61.9% of the sample, while females accounted for the remaining 38.1%. A significant portion of the participants, approximately 64.6%, held a bachelor’s degree, indicating a relatively high level of educational attainment within the sample. Regarding employment status, the vast majority, accounting for 86.9% of respondents, were medical professionals such as doctors. Moreover, the distribution of the sample according to work experience demonstrated a balanced representation across various experience categories, suggesting a diverse range of professional backgrounds and tenure among the participants.

### 3.4. Measures

The measurement of variables in the research study employed rigorous methodologies and established scales. To address the linguistic needs of the targeted Arabic-speaking respondents, the survey instruments were translated from English to Arabic with careful attention to maintaining accuracy and clarity [138]. This translation process was overseen by a language editor to minimize bias and ensure the fidelity of the translated items [139]. Prior to the main study, a pilot study involving 10 medical and administrative workers within public HCOs in Jordan was conducted in June 2022 to assess statistical reliability and internal consistency. Participants were tasked with evaluating the ethical conduct of their supervisors using a 5-point Likert scale, ranging from “strongly agree” to “strongly disagree”.

Ethical leadership was assessed using a 10-item scale developed by Brown et al. [3]. Sample items included statements such as “My supervisor conducts his/her personal life in an ethical manner”. Organizational climate was assessed using 11 items adapted from the work of Nazari et al. [91]. Example items included assertions such as “Taking reasonable risks is acceptable in this organization”. Role overload, a significant factor in employee well-being, was assessed using a set of thirteen items adapted from Jones et al. [78]. Sample items included statements such as “There are too many demands on my time”. Finally, job burnout was evaluated using a 6-item Likert-type scale adopted from Demerouti et al. [140], focusing on emotional exhaustion among medical and administrative workers. Participants indicated their agreement with statements such as “After work, I tend to need more time than in the past to relax and feel better”.

### 3.5. Common Method Bias

Common method bias (CMB) represents a critical concern in research methodology, especially in studies where data is gathered from a single source or relies heavily on self-reported measures, as evident in this investigation. To address potential CMB issues, various mitigation strategies were implemented based on recommendations by Podsakoff et al. [141]. The survey instrument featured a comprehensive consent form outlining the study’s objectives while ensuring respondents the confidentiality and anonymity of their contributions, thus fostering an environment conducive to candid responses [142]. Additionally, Harman’s [143] one-factor test was conducted to evaluate whether a single underlying factor could account for a substantial portion of the observed variance across measured constructs. The results revealed no significant common method bias, with the highest variance explained being approximately 23.3%, thereby enhancing the credibility of the findings. These methodological approaches were instrumental in fortifying the validity and reliability of the study’s outcomes, ensuring robustness in data analysis and interpretation.

## 4. Data Analysis and Results

The data analysis in this study followed a rigorous approach, utilizing partial least squares structural equation modeling (PLS-SEM) in the PLS 4 software. PLS-SEM is renowned for its flexibility and suitability for exploratory research endeavors [144], rendering it an optimal choice for scrutinizing intricate structural models, particularly with small sample sizes [145]. The analysis unfolded in two distinct stages, adhering to established guidelines [146,147]. Initially, the measurement model underwent comprehensive validation to ensure the reliability and validity of the constructs under examination. This process involved scrutinizing validity and reliability measures to confirm the robustness of the measurement model [148]. Concurrently, the structural model was scrutinized to assess the strength and significance of the relationships among the variables [149]. Path coefficients (β), coefficient of determination (*R*^2^), and path significance (*P*) were meticulously evaluated to determine the degree of influence among the variables and the overall fit of the structural model to the data [150]. By employing PLS-SEM, the study rigorously examined the relationships between the variables, ultimately deriving meaningful insights into the underlying mechanisms at play [151].

### 4.1. Validation of the Measurement Model

The validation of the measurement model constituted a crucial step in ensuring the reliability and validity of the constructs evaluated in this study, conducted using PLS-SEM 4. To ascertain the reliability of the measures, essential indicators such as Cronbach’s α, composite reliability, and Average Variance Extracted (AVE) were computed [150]. Internal consistency was confirmed as the values of Cronbach’s α and composite reliability surpassed the threshold of 0.70, indicating acceptable reliability [144,150]. Additionally, the values of rhoA falling between Cronbach’s α and composite reliability further underscored the robustness of the measures [152]. Moreover, the outer model loadings, as presented in Table 2, predominantly exceeded the 0.7 threshold, further reinforcing the reliability of the measures [144]. Furthermore, the assessment of convergent validity, indicated by the AVE values surpassing 0.5 (see Table 2), demonstrated the constructs’ ability to converge on the underlying theoretical concepts [153].

Furthermore, discriminant validity was examined through the Heterotrait–Monotrait (HTMT) ratio, as displayed in Table 3, unveiling values below 0.85, indicating the absence of multicollinearity among the constructs [154]. This analysis validated the discriminant validity of the model, reinforced by the Fornell–Larcker criterion where the square of each variable’s AVE surpassed the intercorrelations (see Table 3), fortifying the validity of the structural model [153]. This thorough examination, combined with the satisfactory psychometric properties of the measurement model [155], provides robust support for the validity and reliability of the constructs evaluated in the study.

### 4.2. Assessment of the Structural Model

The evaluation of the structural model using PLS-SEM enabled rigorous hypothesis testing and the assessment of path coefficients [146,151]. Adhering to guidelines outlined in the PLS-SEM literature, a bootstrapping procedure involving 5000 subsamples was carried out using Smart PLS version 4 software [150,156].

**Figure 2:**
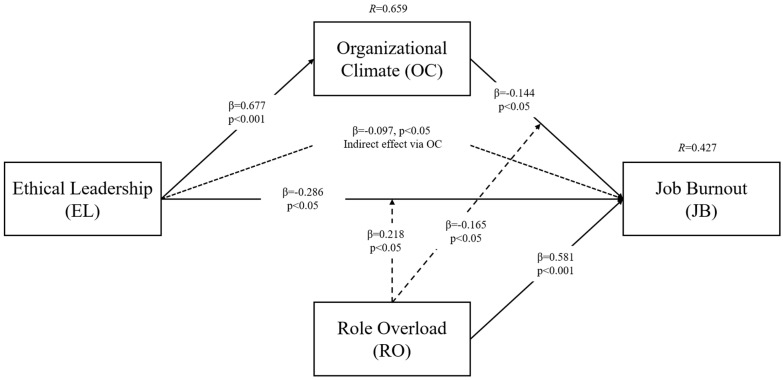
Structural model results.

Before testing the structural model, collinearity underwent rigorous evaluation using the variance inflation factor (VIF), with all VIF values below the threshold of 3.3, indicating no collinearity among the constructs [150]. Subsequently, the bootstrapping procedure was utilized to estimate standard errors and evaluate the significance of parameter estimates [157,158]. The results are illustrated in Figure 2.

The examination of hypotheses revealed significant outcomes as presented in Table 4. H1, proposing a negative relationship between ethical leadership and employee job burnout, garnered full support from the results (β = −0.286, t = 2.497, *p* = 0.013). Likewise, H2, which postulated a positive relationship between ethical leadership and organizational climate, received empirical support (β = 0.677, t = 21.851, *p* = 0.000). H3, suggesting the negative impact of organizational climate on employee job burnout, was also supported (β = −0.144, t = 2.132, *p* = 0.033). Furthermore, the analysis revealed a positive impact of role overload on employee job burnout (β = 0.581, t = 11.491, *p* = 0.000).

The examination of H4, pertaining to the mediation of organizational climate in the relationship between ethical leadership and employee job burnout, yielded statistically significant results (β = −0.097, t = 2.099, *p* = 0.036), providing support for the hypothesis. In addition, H5a, positing the moderation effect of role overload in the relationship between ethical leadership and job burnout, was fully supported (β = 0.218, t = 2.539, *p* = 0.011). Similarly, H5b, proposing the moderation of the impact of role overload in the relationship between organizational climate and employee job burnout, also received empirical support (β = −0.165, t = 2.055, *p* = 0.040), further affirming the intricate interplay among the variables in the structural model.

### 4.3. Explanatory Power of the Structural Model

The explanatory power of the structural model was assessed through examination of the coefficient of determination, or *R*^2^, which provides insights into the proportion of variance explained by the model’s predictors [159]. Utilizing the PLS algorithm in Smart PLS 4 software, *R^2^* values were computed, surpassing the suggested threshold of 0.10, indicating adequate explanatory power [159,160]. As illustrated in Figure 2, the *R*^2^ value for organizational climate was 0.659, signifying that approximately 65.9% of the variance in organizational climate can be explained by the model. Similarly, the *R^2^* value for job burnout was 0.427, indicating that approximately 42.7% of the variance in job burnout is accounted for by the model.

Additionally, the strength of the moderation effect of role overload was rigorously assessed through the calculation of the *f*^2^ statistic, a metric widely employed to gauge the impact size of moderators in structural equation models [161]. Following the formula provided by Cohen [162] and Henseler and Fassott [163], the moderation effect size (*f*^2^) was computed by comparing the *R*^2^ value of the model with the moderator (*R*^2^ model with moderator) to the *R*^2^ value of the model without the moderator (*R*^2^ model without moderator), divided by (1—*R*^2^ model with moderator). This method allows for a comprehensive evaluation of the moderating influence of role overload. In the present study, the obtained *f*^2^ value of 0.614 signifies a robust moderation effect size (Table 5), indicating that role overload significantly influences the relationships among the model’s variables [162,163]. This substantial effect underscores the pivotal role of role overload as a moderator in the structural model, elucidating its profound impact on the dynamics of organizational climate, ethical leadership, and employee job burnout.

**Figure 3:**
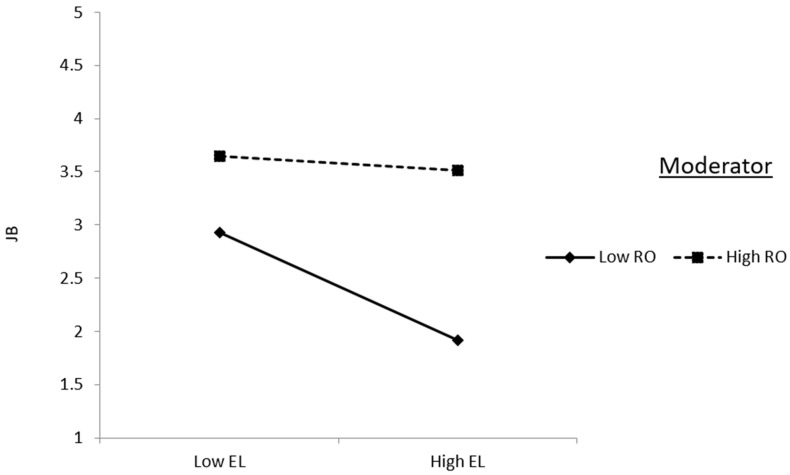
Interaction effect of role overload in the relationship between ethical leadership and job burnout.

**Figure 4:**
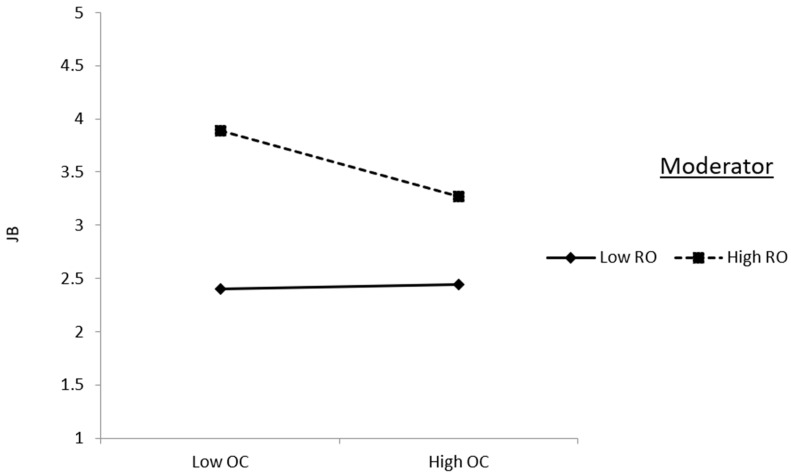
Interaction effect of role overload in the relationship between organizational climate and job burnout.

The moderating effect of role overload (RO) in the structural model is depicted vividly in Figure 3 and Figure 4, shedding light on its intricate interplay with both ethical leadership (EL) and organizational climate (OC) concerning job burnout (JB). In Figure 3, the illustration showcases how role overload serves to diminish the adverse impact of ethical leadership on job burnout. This attenuation implies that despite the presence of ethical leadership, individuals experiencing high levels of role overload may still be susceptible to job burnout, albeit to a lesser extent.

Conversely, Figure 4 elucidates the moderating effect of role overload on the relationship between organizational climate and job burnout. Here, role overload strengthens the negative association between organizational climate and job burnout, suggesting that in environments characterized by supportive organizational climates, the detrimental effects of job burnout may be mitigated, particularly when individuals perceive lower levels of role overload. These figures provide visual representations of how role overload operates as a crucial moderator, influencing the dynamics between ethical leadership, organizational climate, and job burnout in the structural model.

## 5. Discussions and Implications

### 5.1. Discussion of Study Findings

The study’s findings offer significant insights into the dynamics of ethical leadership, organizational climate, role overload, and job burnout, resonating with contemporary research in organizational behavior and leadership. Our first hypothesis posited that ethical leadership negatively correlates with job burnout, a hypothesis that was confirmed by our results. This finding aligns with prior literature emphasizing the critical role of ethical leadership in enhancing employee well-being [1,3,9,21,40]. Leaders who demonstrate ethical behavior and uphold values such as trust, fairness, and integrity can effectively alleviate the risk of job burnout. This is consistent with the notion that ethical leadership fosters a supportive and fair organizational culture that mitigates stress and promotes well-being [3,76,83].

Conversely, our second hypothesis predicted a positive relationship between ethical leadership and organizational climate. Our study confirmed this hypothesis, which is consistent with recent research highlighting the pivotal role of leaders in shaping the broader organizational environment [17,21,24,25,109,112]. Ethical leaders cultivate a positive work environment by setting clear expectations, promoting open communication, and fostering a climate of trust and support. This conducive environment enhances employee well-being and organizational effectiveness [4,47,91,92].

Moreover, our third and fourth hypotheses examined the mediating role of organizational climate in the relationship between ethical leadership and job burnout. The results support this hypothesis, suggesting that a positive organizational climate characterized by trust, collaboration, and shared values significantly mitigates the adverse effects of job burnout [1,16,115]. This finding is in line with previous studies indicating that a supportive climate can enhance employee engagement and retention by reducing stress and promoting a sense of belonging [17,24,50,83,93].

Our final hypothesis explored role overload as a moderator in the relationship between ethical leadership, organizational climate, and job burnout. The study found that role overload exacerbates the detrimental effects of job burnout, especially in environments where organizational support and resources are lacking [44,78,122]. This underscores the nuanced interplay between individual and contextual factors in influencing job burnout [40,115,118]. These findings highlight the importance of organizational interventions aimed at addressing workload management, role clarity, and resource allocation to mitigate burnout risks [31,93,126,131].

Overall, the study’s findings contribute to our understanding of the multifaceted relationships between leadership, organizational climate, role overload, and job burnout. They offer valuable implications for organizational practices and leadership development, particularly in the healthcare sector, where maintaining employee well-being is crucial for both individual and organizational performance. By promoting ethical leadership and cultivating a positive organizational climate, healthcare organizations can better manage role overload and reduce job burnout, ultimately fostering a more resilient and effective workforce.

### 5.2. Theoretical Implications

The study findings yield significant theoretical implications for the fields of organizational behavior, leadership, and employee well-being. Firstly, the confirmed negative relationship between ethical leadership and job burnout aligns with established theoretical frameworks emphasizing the pivotal role of leadership behavior in shaping employee outcomes [7,13,25,40]. Ethical leadership theory posits that leaders who demonstrate integrity, fairness, and ethical conduct foster trust and respect among employees, thereby reducing the risk of burnout [5,6,85,88]. This finding underscores the importance of ethical leadership as a key determinant of employee well-being and organizational effectiveness.

Secondly, the positive relationship between ethical leadership and organizational climate aligns with social exchange theory, which suggests that positive leader behaviors cultivate a supportive organizational climate characterized by trust, cooperation, and mutual respect [36,37]. Leaders who prioritize ethical values and promote transparency and communication contribute to a positive organizational climate that enhances employee satisfaction and engagement [17].

Furthermore, the research contributes significantly to our comprehension of the mechanisms through which ethical leadership influences job burnout. Ethical leadership has been widely acknowledged as a crucial determinant of employee well-being [1,6,13,51]. The concept of organizational climate, highlighted in this study, elucidates how the workplace environment mediates the effects of leadership on employee outcomes [77,85,164]. Drawing from the job demands–resources model, which posits that organizational climate functions as a psychological resource, the research underscores the pivotal role of a positive work environment in mitigating burnout risks [32,34,165,166]. By fostering a supportive atmosphere, organizations can enhance employee resilience and job satisfaction, thereby promoting overall well-being. This emphasizes the significance of creating conducive work environments that prioritize employee welfare.

Lastly, the study’s exploration of role overload as a moderator in the relationship between ethical leadership, organizational climate, and job burnout contributes to our understanding of the boundary conditions that influence leadership effectiveness and employee well-being [44,115,118,119,120]. Role overload theory suggests that excessive job demands and workload can exacerbate the negative effects of leadership behaviors on employee outcomes [40,78,112]. By identifying role overload as a critical factor in the relationship between leadership, organizational climate, and burnout, the study underscores the importance of workload management and resource allocation in promoting employee well-being and organizational effectiveness.

### 5.3. Practical Implications

The study’s findings offer valuable practical implications for organizational leaders and practitioners aiming to improve employee well-being and organizational effectiveness. Firstly, the confirmed negative relationship between ethical leadership and job burnout suggests that organizations should prioritize the cultivation of ethical leadership behaviors among their leaders. This entails providing leadership development programs and training initiatives that focus on promoting integrity, fairness, and transparency in leadership practices. By fostering a culture of ethical leadership, organizations can reduce the risk of employee burnout and promote a positive work environment.

Secondly, the positive relationship between ethical leadership and organizational climate underscores the importance of creating a supportive and inclusive organizational culture. Leaders should actively promote open communication, collaboration, and trust within the organization to foster a positive climate. This can be achieved through initiatives such as regular team meetings, feedback mechanisms, and recognition programs that reinforce ethical values and promote a sense of belonging among employees.

Furthermore, the study’s identification of organizational climate as a mediator in the relationship between ethical leadership and job burnout suggests that interventions aimed at improving the organizational climate can help mitigate the risk of burnout among employees. Organizations should focus on creating a supportive work environment characterized by clear expectations, adequate resources, and opportunities for growth and development. This may involve implementing flexible work arrangements, providing access to employee assistance programs, and fostering a culture of recognition and appreciation.

Lastly, the study’s findings regarding the moderating role of role overload underscore the importance of workload management and resource allocation in mitigating the negative effects of job demands on employee well-being. Organizations should strive to provide employees with the necessary resources, support, and training to effectively manage their workload and navigate competing demands. Implementing flexible work arrangements, providing access to supportive resources, and encouraging work–life balance initiatives can help reduce the risk of role overload and mitigate its negative impact on employee job burnout.

### 5.4. Study Limitations and Suggestions for Future Research

While the research provides valuable insights into the connections among ethical leadership, organizational climate, role overload, and job burnout, it is essential to acknowledge its limitations. Firstly, the reliance on self-reported data introduces the possibility of common method bias and social desirability bias [141]. To enhance the validity of future studies, researchers could incorporate multi-source data collection methods, such as supervisor ratings or objective performance metrics. Additionally, the study’s focus on government hospitals within a specific geographic region restricts the generalizability of the findings to other organizational contexts or sectors. Replicating the study across various industries or cultural settings could offer insights into the robustness of the identified relationships. Furthermore, the predominant use of cross-sectional data limits the ability to establish causality or temporal precedence. Employing longitudinal or experimental research designs would enable a clearer understanding of the causal dynamics between variables over time. Lastly, while the study considers role overload as a moderator, it overlooks other potential moderators, such as individual differences or organizational factors. Future research endeavors should explore additional moderators to delineate the boundary conditions of the relationships under examination [119,120]. Addressing these limitations and charting avenues for future research will contribute to a more comprehensive understanding of the interplay between leadership, organizational climate, and employee well-being.

## Figures and Tables

**Table 1:** Characteristics of the participants.

Demographic Profile	Characteristic	Frequency	Percentage of Respondents
Gender	Male	161	61.9%
	Female	99	38.1%
Marital status	Single	83	31.9%
	Married	177	68.1%
Education Level	Bachelor	168	64.6%
	Diploma or below	65	25.0%
	Postgraduate	27	10.4%
Experience	Between 10 and 15 years	59	22.7%
	More than 15 years	74	28.5%
	Between 5 and less than 10 years	63	24.2%
	Below 5 years	64	24.6%
Position	Administrative staff	34	13.1%
	Medical professionals	226	86.9%
	Total	260	100.0%

**Table 2:** Reliability and validity of the measurement model.

Factors	Indicators	Outer Loadings	VIF	Cronbach Alpha Values	RhoA	CR	AVE
Ethical Leadership				0.955	0.958	0.961	0.714
	EL1	0.771	1.677				
	EL2	0.802	1.512				
	EL3	0.799	1.444				
	EL4	0.868	1.594				
	EL5	0.908	1.478				
	EL6	0.848	1.562				
	EL7	0.898	2.227				
	EL8	0.856	2.424				
	EL9	0.855	2.012				
	EL10	0.835	2.316				
Organizational Climate				0.928	0.931	0.940	0.636
	OC1	0.914	2.472				
	OC2	0.705	2.023				
	OC3	0.780	2.669				
	OC4	0.783	2.019				
	OC5	0.904	2.319				
	OC6	0.931	2.186				
	OC7	0.909	2.719				
	OC8	0.820	2.006				
	OC9	0.867	2.338				
	OC10	0.920	2.735				
	OC11	0.844	2.008				
Job Burnout				0.851	0.888	0.882	0.522
	JB1	0.755	1.586				
	JB2	0.798	1.568				
	JB3	0.825	1.854				
	JB4	0.779	1.717				
	JB5	0.715	1.461				
	JB6	0.868	1.376				
Role Overload				0.865	0.875	0.894	0.589
	RO1	0.762	2.091				
	RO2	0.831	2.957				
	RO3	0.719	2.990				
	RO4	0.791	1.870				
	RO5	0.751	1.587				
	RO6	0.835	1.592				
	RO7	0.868	1.947				
	RO8	0.852	1.726				
	RO9	0.745	2.141				
	RO10	0.768	1.282				
	RO11	0.834	2.622				
	RO12	0.761	1.616				
	RO13	0.704	2.406				

**Table 3:** Discriminant validity of constructs.

Factors	1	2	3	4
1. Ethical leadership	***0.845***	0.279	0.174	0.757
2. Job burnout	−0.267	***0.723***	0.654	0.226
3. Role overload	−0.125	0.609	***0.699***	0.164
4. Organizational climate	0.712	−0.212	−0.062	***0.797***

Note: Values in bold and italic denote the square of each variable’s AVE, where the lower values denote the surpassed intercorrelations and the upper-level values denote the HTMT values.

**Table 4:** Results of structural model hypotheses testing.

Path	Hypothesis	Standardized Path Coefficients	t-Values	Confidence Intervals		Decision
				Lower 2.5%	Upper 97.5%	
Direct effects						
Ethical leadership → job burnout	H1	−0.286 *	2.497	−0.508	−0.060	Supported
Ethical leadership → organizational climate	H2	0.677 ***	21.851	0.621	0.742	Supported
Organizational climate → job burnout	H3	−0.144 *	2.132	−0.285	−0.017	Supported
Indirect effect						
EL → (OC) → JB	H4	−0.097 *	2.099	−0.197	−0.012	Supported
Interaction effects						
EL_X_RO → job burnout	H5a	0.218 *	2.539	0.027	0.374	Supported
OC_X_RO → job burnout	H5b	−0.165 *	2.055	−0.303	−0.025	Supported

Note: In our study, EL_X_RO represents the interaction between ethical leadership and role overload, while OC_X_RO represents the interaction between organizational climate and role overload. Significance levels are denoted as follows: * indicates statistical significance at *p* < 0.050 and *** indicates statistical significance at *p* < 0.001.

**Table 5:** Strength of moderating effects.

Moderating Variable	*R* ^2^		*F* ^2^	Effect Size
	Included	Excluded		
Role overload	0.427	0.075	0.614	Strong

Note: In our study, *R*^2^_included represents the coefficient of determination (*R*^2^) of the model with the moderator (role overload), and *R*^2^_excluded represents the *R*^2^ of the model without the moderator.

## Data Availability

The raw data supporting the conclusions of this article will be made available by the authors upon request.
